# Clinical observation of biomimetic mineralized collagen artificial bone putty for bone reconstruction of calcaneus fracture

**DOI:** 10.1093/rb/rbx033

**Published:** 2018-02-08

**Authors:** Yong-Xiong Pan, Guang-Gang Yang, Zhong-Wan Li, Zhong-Min Shi, Zhan-Dong Sun

**Affiliations:** 1 Department of Foot and Ankle Orthopedics, Guangzhou Orthopedic Hospital, Guangzhou 510045, China and; 2 Department of Foot and Ankle Orthopedics, Shanghai Sixth People’s Hospital, Shanghai 200233, China

**Keywords:** calcaneus fracture, mineralized collagen artificial bone putty, bone substitute, bone grafting

## Abstract

This study investigated clinical outcomes of biomimetic mineralized collagen artificial bone putty for bone reconstruction in the treatment of calcaneus fracture. Sixty cases of calcaneal fractures surgically treated with open reduction and internal fixation in our hospital from June 2014–2015 were chosen and randomly divided into two groups, including 30 cases treated with biomimetic mineralized collagen artificial bone putty as treatment group, and 30 cases treated with autogenous ilia as control group. The average follow-up time was 17.2 ± 3.0 months. The results showed that the surgery duration and postoperative drainage volume of treatment group were significantly lower than control group; there were no statistically significant differences in the fracture healing time, American Orthopaedic Foot and Ankle Society scores at 3 and 12 months after surgery, Böhler’s angle, Gissane’s angle and height of calcaneus between the two groups. There were no significant differences in wound complication and reject reaction between the two groups, while significant difference in donor site complication. As a conclusion, the implantation of biomimetic mineralized collagen artificial bone putty in the open reduction of calcaneal fracture resulted in reliable effect and less complications, which is suitable for clinical applications in the treatment of bone defect in calcaneal fractures.

## Introduction

Calcaneus fracture is very common in clinic, accounting for about 2% of systemic fracture and 60% of all tarsal fractures [1]. Calcaneus fracture types of Sanders II, III and IV are usually treated with open reduction and internal fixation. Calcaneus is mainly composed of cancellous bone and its facture is often caused by high falling injury, traffic accident and other high-energy injuries, resulting in different degrees of collapse of joint surface and compression of spongy bone; pry poking reduction of the articular surface during surgery will cause further compression and loss of the cancellous bone. Therefore, bone grafting is necessary in serious bone defect area to support bone on restored subtalar joint facet, and prevent postoperative articular surface collapse which may cause failure of the restoration. Moreover, the implanted bone can promote the healing of bone fracture [2, 3].

Ideal bone graft substitutes should possess the following properties: excellent biocompatibility; beneficial for the ingrowth of blood vessel and osteoblast; comfortable biodegradability; osteoconductivity and osteogenesis; extensive sources and no risk of rejection [4]. Autografts, allografts, and many types of artificial bone grafts have been used for bone repair in the treatment of bone fractures with bone defects. However, there are many drawbacks in the use of these bone grafts or biomaterials. For example, autografting results in new trauma and bone defects [5]; allografts or xenografts have high risk of immunologic rejection or infection [6, 7]; bioceramics are non-degradable and only have limited osteoconductivity [8]; synthetic biopolymers (e.g. polylactic acid, polyglycolic acid, etc.) produce acidic degradation products that adverse to bone regeneration [9]. Moreover, most of the clinical available bone grafts are particles or blocks without moldability, thus being not suitable for bone repair of commonly irregular defects for calcaneus fractures.

**Figure 1:**
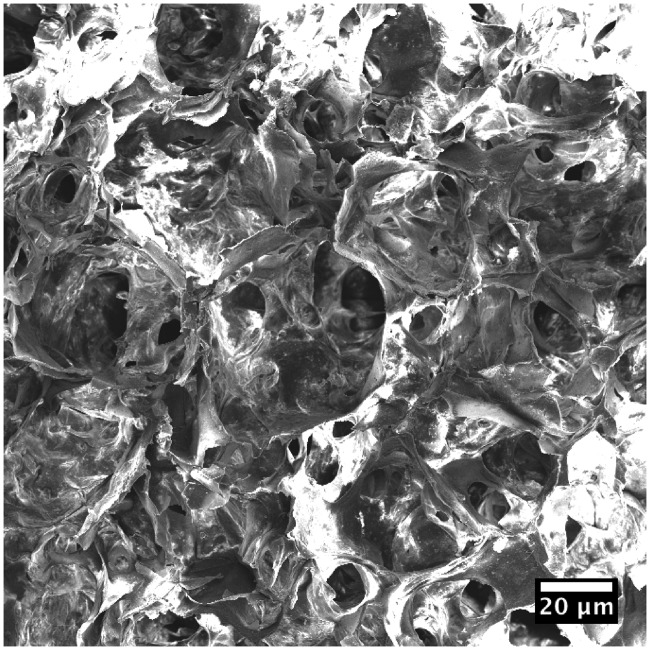
Scanning electron microscopic observation of the mineralized collagen artificial bone putty

Artificial mineralized collagen is a biomimetic bone graft that composed of orderly arranged collagen and nano-sized hydroxyapatite (HA), possessing similar composites and microstructure to the natural bone matrix [10–12]. This biomimetic material has been commercialized and has been applied in many clinical areas related to bone defect repair, including orthopedics, stomatology, neurosurgery and so on [13–16]. The artificial mineralized collagen is biodegradable and has porous microstructure with high porosity and interconnected pores (Fig. 1), which provide sufficient space for osteocytes ingrowth and new bone tissue creeping substitution [10]. Due to the swelling property of the collagen, mineralized collagen could be mixed with water or blood to become a form of putty with good moldability and injectability [17, 18]. So, such mineralized collagen putty is suitable for irregular defect filling and repair for calcaneus fracture. In the present clinical observation, we studied clinical outcomes of biomimetic mineralized collagen artificial bone putty for calcaneus fracture treated with open reduction and internal fixation compared with autologous iliac bone graft.

## Methods

### General data

This study was based on a prospective, randomized and single blind clinical design. A total of 60 patients with calcaneal fracture in our hospital from June 2014 to June 2015 were selected and all the patients met the diagnostic criteria of calcaneal fracture, that is, X-ray and CT indication of calcaneal fracture and obvious swelling and pain occurred at the heel. Inclusive criteria: (i) closed calcaneal fracture of Sanders II, III and IV types; (ii) bone defects >2 cm^3^ in open reduction of fracture; (iii) patients who signed informed consent voluntarily. Exclusion criteria: (i) pathological fractures of calcaneal; (ii) old calcaneal fractures; (iii) open calcaneal fractures; (iv) systemic multiple fractures; (v) patients with abnormal liver and kidney function or with blood coagulation abnormalities who cannot tolerate surgery; (vi) patients were suffered from traumatic arthritis or rheumatoid arthritis at ankle and foot; (vii) patients who were unwilling to sign informed consent. As shown in Table 1, there were 30 cases in the treatment group, including 27 males and 3 females aged 25–58-years old with an average of 41.8 ± 10-years old, among which there were 9 cases of Sanders Type II fractures, 18 cases of Sanders Type III fractures and 3 cases of Sander type IV fractures. The control group consisted of 30 cases, 28 males and 2 females, aged 2 4–56-years old with an average of 41.7 ± 7.7 years old, among which 7 cases were Sanders Type II fractures, 19 cases were Sanders Type III fractures and 4 cases were Sanders Type IV fractures. The differences of sex ratio, mean age and Sanders fracture classification between the two groups were not statistically significant, indicating they were comparable. The study was approved by the ethics committee of the hospital.

**Table 1:** General data of the patients

Group	*n*	Age	Gender		Cases (Sanders Classification II/III/IV)
			Male	Female	
Treatment	30	41.8 ± 10.0	27	3	9/18/3
Control	30	41.7 ± 7.7	28	2	7/19/4
Statistic		*t* = 0.087	$\chi^{2}$ = 0.000		$\chi^{2}$ = 0.421
		*P* > 0.05	*P* > 0.05		*P* > 0.05

### Therapy

**Figure 2:**
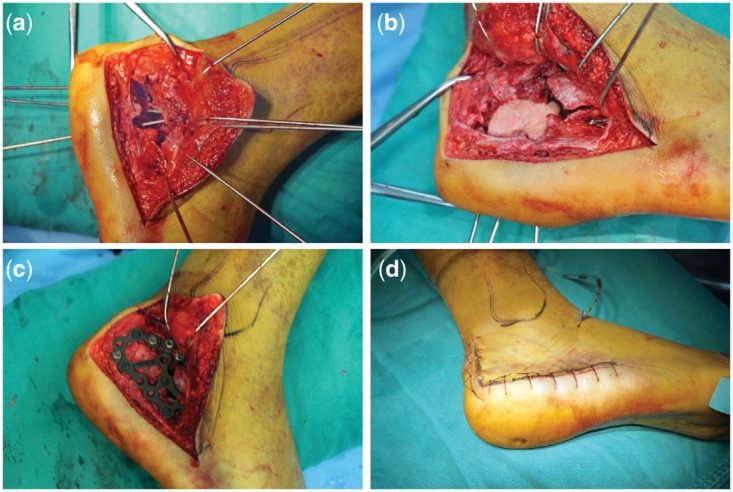
Critical steps of the treatment of a calcaneus fracture with mineralized collagen bone putty. (**a**) Exposure of bone defect of the calcaneus fracture; (**b**) implantation of the mineralized collagen artificial bone putty; c. fixation of the treated calcaneus with calcaneal anatomic locking plates; d. appearance at the completion of the surgery)

The surgeries of the two groups were performed by the same doctors. After admission, the patients were treated with detumescence and pain relief treatment, and the surgery was performed after full detumescence of the injured foot. The operation was performed by epidural anesthesia with an electric tourniquet on the ipsilateral thigh. The incision was made with the “L” standard approach outside the calcaneus, and the full-thickness skin was incised with an upward sharp peeling of full-thickness flap. Three to five Kirschner with a diameter of 2.0 mm were driven into the talus and cuboid, upward bending to expose the subtalar articular surface. The ruptured bony cortex of the outboard calcaneus was open to expose the subsided articular surface bone block, and poking reduction was performed to restore the articular surface. Then the calcaneal height, Böhler’s angle and Gissane’s angle were restored and the internal and valgus calcaneus deformity were corrected with the temporary fixation of Kirschner. Finally, the C-arm perspective was used to check whether the reset was satisfactory. After the reset, bone grafting was performed where the central triangular defects of the calcaneus were >2 cm^3^; the ipsilateral iliac bone grafting was used in the control group while biomimetic mineralized collagen artificial bone putty (produced by Beijing Allgens Medical Science and Technology Co., Ltd.) was used in the treatment group. The modulation of the mineralized collagen putty during the surgery was the same to previous clinical studies [18]. Finally, both of the two groups were treated with calcaneal anatomic locking plates to fix, negative pressure drainage devices, wound suture and dressing before the completion of the surgery. Figure 2 shows key steps of the surgery.

### Observational indexes

The surgery duration, postoperative drainage volume, fracture healing time, Böhler’s angle, Gissane’s angle and calcaneus height after surgery, as well as postoperative wound complications, rejection and donor site complications were compared between patients of the two groups. The injured foot functional scores for 3 and 12 months after operation, according to the scoring system of American Orthopaedic Foot and Ankle Society (AOFAS) (totally 100 points), includes pain, function, autonomic activities, walking, gait, ankle stability, alignment, and so on, where 90–100 is excellent, 75–89 is good, 50–74 is general and <50 is bad.

### Statistical analysis

SPSS 18.0 was used for statistical analysis. All enumeration data were reported as frequency and comparison between groups was performed with $\chi^{2}$ test. The measurement data were reported as the mean ± standard deviation, and independent sample *t* test with an inspection level of *a* = 0.05 was used to perform comparison between groups.

## Results

The operation duration and postoperative drainage volume of the treatment group were lower than those of the control group and the difference was statistically significant (*P* < 0.05). There was no significant difference (*P* > 0.05) in the fracture healing time between the two groups, as shown in Table 2. The difference of Böhler’s angle, Gissane’s angle and calcaneus height between the two groups was not statistically significant (*P* > 0.05), as shown in Table 3. There was no significant difference (*P* > 0.05) in AOFAS scores between the two groups at 3 or 12 months after the surgeries, but the score of the 12 months was much higher than that of the 3 months, as shown in Table 4. The obvious improvement of the AOFAS scores indicated significant recovery of the calcaneus fractures. No significant difference (*P* > 0.05) was found in the wound complications and reject reaction of the implants between the two groups, while the donor site complications were significantly different (*P* < 0.05) between the two groups, as shown in Table 5. 60 patients in the two groups were followed up for a long time and no adverse events occurred during the follow-up period.

**Table 2:** Clinical indexes comparison between the treatment and control groups

Group	N	Operation duration (min)	Postoperative drainage volume (ml)	Fracture healing time (weeks)
Treatment	30	75.90 ± 4.75	231.33 ± 44.16	10.03 ± 1.73
Control	30	86.93 ± 5.26	266.00 ± 54.81	9.80 ± 1.75
Statistic		*t* = −8.527	*t* = −2.698	*t* = 0.519
		*P* = 0.000	*P* = 0.009	*P* = 0.606

**Table 3:** Treatment effects between the treatment and control groups

Group	Parameter	Preoperation	Postoperation	12 months postoperation
Treatment	Böhler’s angle (°)	4.70 ± 6.52	31.40 ± 3.61^a^	30.27 ± 3.35^a^
	Gissane’s angle (°)	137.17 ± 8.83	117.07 ± 5.70^a^	116.17 ± 5.36^a^
	Calcaneus height (mm)	39.13 ± 3.26	48.50 ± 3.22^a^	47.77 ± 2.93^a^
Control	Böhler’s angle (°)	3.77 ± 7.15	30.87 ± 3.91^a^	29.70 ± 3.11^a^
	Gissane’s angle (°)	137.70 ± 7.62	118.40 ± 5.84^a^	117.40 ± 4.70^a^
	Calcaneus height (mm)	38.20 ± 2.94	47.23 ± 2.86^a^	46.47 ± 2.52^a^

^a^Indicates that there was statistically significant difference compared with preoperation.

**Table 4:** AOFAS scores between the treatment and control groups

Group	*n*	3 months	12 months
Treatment	30	65.23 ± 5.72	88.37 ± 3.61
Control	30	66.03 ± 4.20	88.37 ± 4.74
Statistic		*t* = −0.617	*t* = 1.840
		*P* = 0.539	*P* = 0.071

**Table 5:** Donor site complications between the treatment and control groups

Group	*n*	Wound complications (cases)	Rejection (cases)	Donor site complications (cases)
Treatment	30	2	0	0
Control	30	2	0	8
Statistic		$\chi^{2}$ = 0.000		$\chi^{2}$ = 7.067
		*P* = 1.000		*P* = 0.008

Typical Case 1: A 41-year-old male patient with injuries by falling leading to fractures of the right calcaneus was treated with incision reduction and internal fixation. 2 cm^3^ mineralized collagen bone putty was used in the surgery.

Typical Case 2: A 52-year-old male patient with injuries by falling leading to fractures of the left calcaneus was treated with incision reduction and internal fixation. 2 cm^3^ mineralized collagen bone putty was used in the surgery.

Typical Case 3: A 36-year-old male patient with injuries by falling leading to fractures of the right calcaneus was treated with incision reduction and internal fixation. 2 cm^3^ mineralized collagen bone putty was used in the surgery.

**Figure 3:**
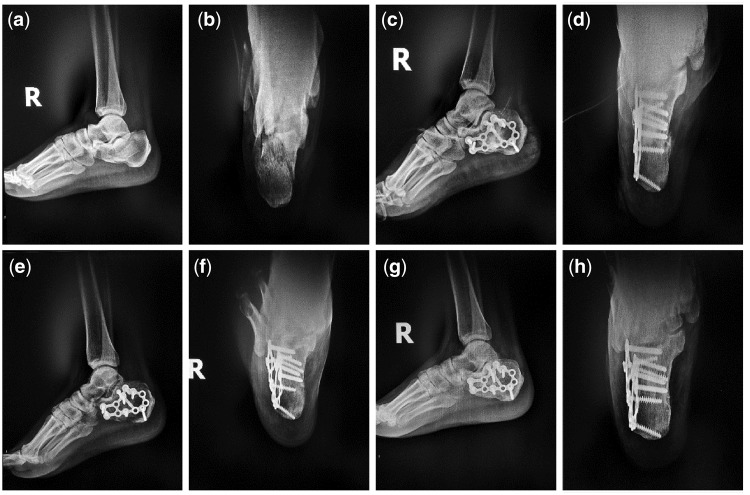
Plain radiographs of the first typical case. (**a** and **b**) Preoperative lateral and anteroposterior films; (**c** and **d**) immediately postoperative lateral and anteroposterior films; (**e** and **f**) 3 months postoperative lateral and anteroposterior films; (**g** and **h**) 12 months postoperative lateral and anteroposterior films)

**Figure 4:**
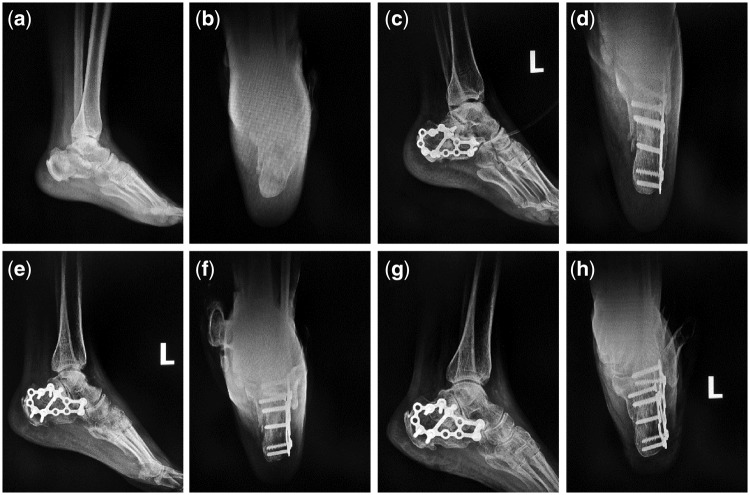
Plain radiographs of the second typical case. (**a** and **b**) Preoperative lateral and anteroposterior films; (**c** and **d**) immediately postoperative lateral and anteroposterior films; (**e** and **f**) 3 months postoperative lateral and anteroposterior films; (**g** and **h**) 12 months postoperative lateral and anteroposterior films)

**Figure 5:**
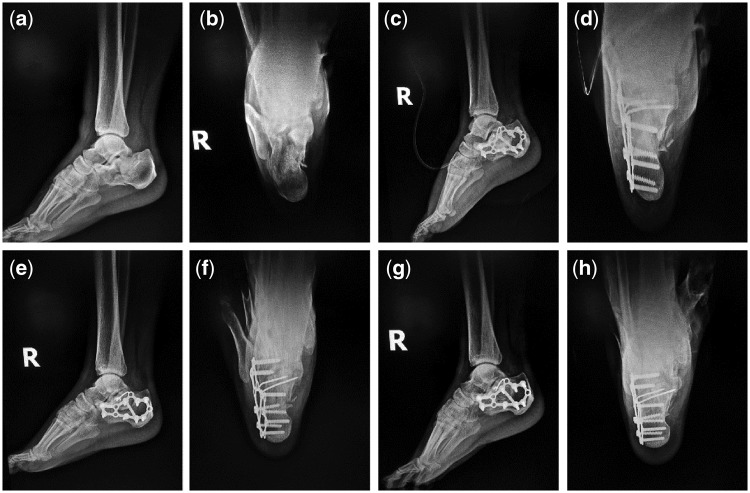
Plain radiographs of the third typical case. (**a** and **b**) preoperative lateral and anteroposterior films; (**c** and **d**) immediately postoperative lateral and anteroposterior films; (**e** and **f**) 3 months postoperative lateral and anteroposterior films; (**g** and **h**) 12 months postoperative lateral and anteroposterior films)

For the three typical cases, Böhler’s angle, Gissane’s angle and Calcaneus height of the treated calcaneus fractures were obviously improved (Tables 6–8). For each case, AOFAS score of 12 months post-operation was much higher than that of 3 months (Table 9). According to plain radiographs of each case (Figs 3–5), significant recovery of the calcaneus fracture could be observed. Pre-operation: calcaneus fractured and collapsed to be flat. Subtalar joint facet was involved (Figs 3a, b, 4a, b, 5a and b). Postoperation: the fracture line aligned well after the calcaneus operation without abnormalities on internal fixations (Figs 3c, d, 4c, d, 5c and d). Three months postoperation: the fracture line aligned well with bony callus formation, and the internal fixations were stable (Figs 3e, f, 4e, f, 5e and f). Twelve months postoperation: the fracture line aligned well and blurred, the fracture healed very well, and the internal fixations were stable (Figs 3g, h, 4g, h, 5g and h).

**Table 6:** Treatment effects of typical Case 1

Observation time	Böhler’s angle (°)	Gissane’s angle (°)	Calcaneus height (mm)
Preoperation	−9	137	39
Postoperation	38	110	46
3 months postoperation	37	109	45
12 months postoperation	37	109	45

**Table 7:** Treatment effects of typical Case 2

Observation time	Böhler’s angle (°)	Gissane’s angle (°)	Calcaneus height (mm)
Preoperation	5	135	39
Postoperation	38	119	48
3 months postoperation	37	116	48
12 months postoperation	37	116	48

**Table 8:** Treatment effects of typical Case 3

Observation time	Böhler’s angle (°)	Gissane’s angle (°)	Calcaneus height (mm)
Preoperation	0	128	40
Postoperation	23	118	44
3 months postoperation	23	118	44
12 months postoperation	23	118	44

**Table 9:** AOFAS scores of the three typical Cases at 3 and 12 months postoperation

Observation time	Case 1	Case 2	Case 3
3 months postoperation	65	63	63
12 months postoperation	87	85	90

## Discussion

There is always a certain degree of bone defect after the reduction of intra-articular fracture of calcaneus. Sanders III or IV fracture may produce 7–15 cm^3^ bone defect after the reduction. So, we consider bone grafting is necessary in the treatment. Bone grafting in the bone defect area can provide mechanical support for the subsided subtalar posterior articular surface and prevent nonunion of bone defect or secondary collapse of the calcaneus. Bone grafting can play a role in bone induction and bone conduction, promote early healing of calcaneal fractures [19, 20], and fill defect area to prevent accumulation of blood and infection. When compared with the treatment without bone grafting, bone grafting treatment of the calcaneal fracture could make the patient to walk with load and do functional exercise earlier. Such acceleration is positive to function rehabilitation and living quality improvement [21]. Moreover, filling the bone defects by bone grafting could prevent hematocele, and reduce infection rate [22]. Nowadays, bone graft materials used in clinic include autogenous bone, allograft bone and synthetic bone graft substitute materials. Autogenous bone graft, especially iliac, has good biocompatibility, no immune rejection, osteogenic induction ability and other natural advantages [23–26]; however, it also has some disadvantages due to the limited materials, additional surgical trauma, prolonged operation time, increased bleeding and sometimes infection, pain, numbness and other complication [27]. Although the source of allograft bone is wide, there are risks that it may spread disease and the common rejection will lead to wound exudate and poor healing [28]; moreover, the healing is slow because there is no osteoinductive effect. The insufficient of the former two promoted the emergence and development of synthetic bone graft material and a good synthetic bone transplantation substitute material not only need to have good bone conduction, bone inducing activity, biocompatibility and appropriate degradation rate, but also play a supporting role through rapid solidification during surgery, without the risk of rejection.

Biomimetic artificial mineralized collagen is a synthetic HA and collagen-based material developed in recent years, highly simulating human bone tissue [12, 29, 30], with the same chemical composition and microscopic structure as human natural bone matrix, which can provide a good microenvironment for bone cell activities to guide bone tissue regeneration and has been widely used in orthopedics, stomatology, neurosurgery and plastic surgery [13, 16, 31, 32]. Its excellent bone defect repairmen effect has been proved. Biomimetic mineralized collagen artificial bone putty can turn to a paste quickly when mixed with water and stirred for about 2 min during surgery, which can fully fill the bone defect area [18]. However, the autogenous iliac bone grafting takes a long time with a limited amount, and is not sufficient for implantation and cannot perfectly fill in the bone defect area as biomimetic mineralized collagen artificial bone putty, calcium sulfate and other artificial implants do. As a result, the use of biomimetic mineralized collagen artificial bone putty can effectively shorten the operation time and fill the bone defect area better thus decrease postoperative drainage, as seen in this study. Biomimetic artificial mineralized collagen with high porosity and pore size ranged from about 100–300 microns, simulates the microporous structure of cancellous bone and can stimulate the adsorption of own proteins and the growth of capillaries, offering a natural environment for bone growth; and it has good biocompatibility, osteoconductivity and osteoinductivity [3, 33]. Biomimetic mineralized collagen artificial bone putty can be completely biodegradable *in vivo* in about 3–6 months, which is roughly in line with the healing time of fracture and beneficial for the creeping substitution of the fracture defect, so there was no obvious difference in fracture healing time compared with autogenous iliac graft in this study. At the same time, there is no rejection of implanted biomimetic mineralized collagen artificial bone material in this study and most of the patients healed well and had fewer complications.

This study shows that biomimetic mineralized collagen artificial material is a kind of artificial bone material with good biocompatibility and osteoinduction which can quickly fill irregular bone defect area and be completely absorbed; and the treatment of calcaneal fracture with locking plate can achieve satisfactory clinical results.

*Conflict of interest statement*. None declared.
